# Monitoring of Grouting Compactness in a Post-Tensioning Tendon Duct Using Piezoceramic Transducers

**DOI:** 10.3390/s16081343

**Published:** 2016-08-22

**Authors:** Tianyong Jiang, Qingzhao Kong, Wenxi Wang, Linsheng Huo, Gangbing Song

**Affiliations:** 1 College of Civil Engineering and Architecture, Changsha University of Science and Technology, Changsha 410114, China; tianyong_jiang@foxmail.com; 2 Department of Mechanical Engineering, University of Houston, Houston, TX 77004, USA; qkong@uh.edu; 3 College of Civil Engineering, Hunan University, Changsha 410082, China; wangwenxi_hnu@sina.com; 4 School of Civil Engineering, Dalian University of Technology, Dalian 116024, China; lshuo@dlut.edu.cn

**Keywords:** lead zirconate titanate (PZT), smart aggregate, reinforced concrete, post-tensioning tendon duct, grout, grouting compactness

## Abstract

A post-tensioning tendon duct filled with grout can effectively prevent corrosion of the reinforcement, maintain bonding behavior between the reinforcement and concrete, and enhance the load bearing capacity of concrete structures. In practice, grouting of the post-tensioning tendon ducts always causes quality problems, which may reduce structural integrity and service life, and even cause accidents. However, monitoring of the grouting compactness is still a challenge due to the invisibility of the grout in the duct during the grouting process. This paper presents a stress wave-based active sensing approach using piezoceramic transducers to monitor the grouting compactness in real time. A segment of a commercial tendon duct was used as research object in this study. One lead zirconate titanate (PZT) piezoceramic transducer with marble protection, called a smart aggregate (SA), was bonded on the tendon and installed in the tendon duct. Two PZT patch sensors were mounted on the top outside surface of the duct, and one PZT patch sensor was bonded on the bottom outside surface of the tendon duct. In the active sensing approach, the SA was used as an actuator to generate a stress wave and the PZT sensors were utilized to detect the wave response. Cement or grout in the duct functions as a wave conduit, which can propagate the stress wave. If the cement or grout is not fully filled in the tendon duct, the top PZT sensors cannot receive much stress wave energy. The experimental procedures simulated four stages during the grout pouring process, which includes empty status, half grouting, 90% grouting, and full grouting of the duct. Experimental results show that the bottom PZT sensor can detect the signal when the grout level increases towards 50%, when a conduit between the SA and PZT sensor is formed. The top PZT sensors cannot receive any signal until the grout process is completely finished. The wavelet packet-based energy analysis was adopted in this research to compute the total signal energy received by PZT sensors. Experimental results show that the energy levels of the PZT sensors can reflect the degree of grouting compactness in the duct. The proposed method has the potential to be implemented to monitor the tendon duct grouting compactness of the reinforced concrete structures with post tensioning.

## 1. Introduction

With the widespread use of reinforced concrete structures using post-tensioning tendons, there is a potential problem during construction: the tendon duct may not be fully grouted. This results in voids or inefficient protection for the steel tendons. Over the long term, water may enter the tendon ducts in the voids, resulting in corrosion of the tendons [1,2]. Therefore, to guarantee the durability of reinforced concrete structures with post-tensioning tendons, the tendon ducts must be completely filled with concrete to avoid water intrusion and corrosion of steel tendons.

Non-destructive inspection of ducts plays a critical role in the quality assurance of construction [3]. Non-destructive testing (NDT) techniques, including ultrasonic, impact-echo (IE), ground penetrating radar (GPR), are presented by Muldoon et al. for the investigation of tendon ducts [4]. Wang et al. applied ultrasonic method to detect the grouting quality of pre-stressed corrugated ducts [5]. The defects were set in the duct, including empty duct, 1/4 grouting, half grouting, 3/4 grouting and full grouting. The results showed that the quality of duct grouting can be effectively evaluated by ultrasonic detection. Krause et al. described the use of an ultrasonic array system combined with 3D reconstruction computation [6]. This system was applied to the examination of transversal ducts in a bridge plate. Schickert et al. adopted the synthetic aperture focusing technique (SAFT) of ultrasonic reconstruction to detect the tendon ducts containing faults [7]. Muldoon et al. tested a series of standard concrete beams with plastic P-T (post-tensioning) tendon ducts at the University of Edinburgh using a range of NDT techniques including impact-echo (I-E), stack imaging of spectral amplitudes based on the impact-echo (SIBIE) and ultrasonic tomography [4]. Yamada et al. developed the Stack Imaging of Spectral Amplitudes Based on Impact-Echo (SIBIE) procedure to improve the impact-echo method [2]. It was demonstrated that the non-grouted tendon-duct can be identified with reasonable accuracy by the SIBIE procedure. Maierhofer showed the recent applications of ground penetrating radar (GPR) at frequencies from 500 MHz to 2.5 GHz for detection of different defects in concrete structures [8]. The technique is well suited for locating tendon ducts at depths up to 50 cm, and to detecting voids and detachments. Another application of GPR is presented by Bungey et al., who adopted the GPR based method to monitor the grouting deficiencies in the suggested non-metallic ducts [9]. In general, the existing research on detection of grout quality requires bulky tools and equipment, and the reported methods are not suitable for real time monitoring, which motivates us to develop a low-cost and real-time method to monitor the grouting process and to evaluate the grouting compactness.

With the advantages of availability in different shapes, high bandwidth, low price, and the ability to be employed as an actuator and sensor simultaneously, the lead zirconate titanate (PZT)-based active sensing approach has been widely recognized as one of the most promising structural health monitoring (SHM) techniques [10,11,12,13,14,15,16,17]. In additional, a numerical modelling of PZT-based structural health monitoring has also been studied [18]. To further use PZT material for practical applications of structural damage evaluation, Chalioris et al. proposed a wireless admittance monitoring system to detect damage to reinforced concrete steel bars [19]. Due to the fragility of PZT material, Song et al. developed a PZT-based multi-functional smart aggregate (SA), which can be deployed in a distributed fashion, for SHM of concrete structures in recent years [20,21,22]. A proposed wavelet packet energy index was verified to be effective in the experimental study on CFST (concrete-filled steel tube) specimens with artificially simulated debonding using PZT patches and SAs [23,24]. Wu et al. performed an investigation to detect debonding in reinforced concrete structures utilizing built-in piezoelectric discs as sensors and actuators in a pitch-catch mode [25,26]. Qin et al. developed an active sensing approach using smart aggregates (SAs) to detect the initiation and to monitor the development of bond-slip of steel plate concrete beams [27]. Chalioris et al. applied impedance-based technology and analyzed the smart aggregates’ admittance signatures that can successfully evaluate the damage levels in the shear-critical reinforced concrete beam [28]. The SAs can be utilized as both actuator and sensor by taking advantage of the piezoelectricity of piezoceramic material. Hence, the proposed smart aggregates are capable of performing comprehensive monitoring of concrete structures, including early age strength monitoring [29], impact detection and evaluation [20], and structural health monitoring [21].

This paper proposes a stress wave-based active sensing approach using piezoceramic transducers to monitor the grouting compactness of post-tensioning tendon duct for reinforced concrete structures. The proposed method is capable of monitoring the grouting compactness in real time. In the proposed method, one PZT-based SA, which was bonded on the tendon, was utilized as an actuator. Meanwhile, three waterproofed PZT patches used as sensors were mounted on the top and bottom surfaces (outside) of a tendon duct before casting. Grout in the duct functions as the wave conduit which allows the passage of the stress wave. If the duct is 100% filled with the grout, the stress wave generated by the SA will propagate to reach both the top and bottom surfaces of the tendon duct via the grout. However, without the presence of any grout, the stress wave will fail to reach either the top or the bottom of the duct. With partially filled grout, the stress wave may reach or fail to reach one or both of the top or bottom surface of the duct. In addition, the wavelet packet-based energy analysis was adopted in this research to compute the total signal energy received by PZT sensors. In this research, four different grout stages, including empty, half grouting, 90% grouting, and full grouting, were experimentally studied. In order to simulate these four situations in one specimen, the grout was poured to reach each stage in the increment of five days. The eventual 100% grouting condition can be confirmed when the PZT sensors on the top surface of the duct detect the stress wave and report high levels of energy.

## 2. Piezoceramic Transducers

### 2.1. Compressive Mode PZT Transducers

**Figure 1:**
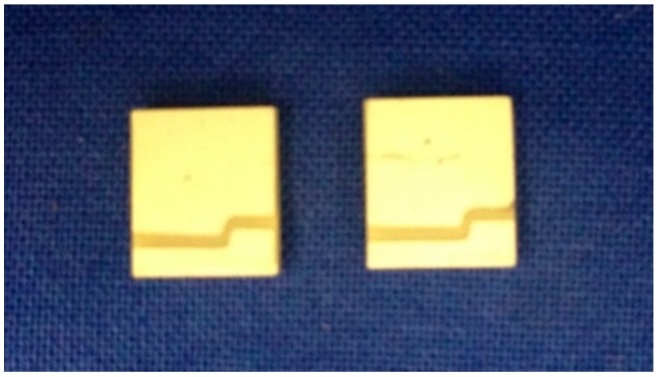
The photo of two PZT patches.

**Figure 2:**
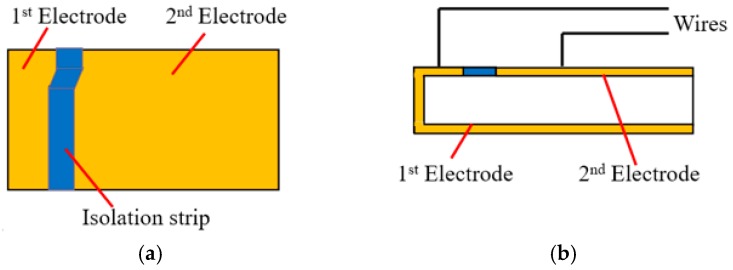
The two drawings of the PZT patch: (**a**) Top view; (**b**) Front view.

**Figure 3:**
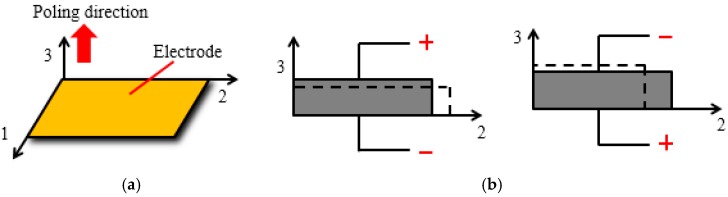
Schematic of PZT patch with compressive mode: (**a**) 3D view; (**b**) Front view.

Piezoelectric material generates electric charge when subjected to a stress or strain (the direct piezoelectric effect) and also produces a stress or strain when an electric field is applied in the poled direction (the converse piezoelectric effect). Lead zirconate titanate (PZT) is one of the most commonly used piezoelectric materials and can be cut into small sizes as sensors. An often used shape is the patch, as shown in Figure 1. The structure of the PZT patch is shown in Figure 2. When PZT patch is in compression mode, it can expand and shrink along the poling direction corresponding to the applied voltage, as shown in Figure 3. In recent years, PZT sensors have been employed in many applications of structural health monitoring. Wang et al. monitored the bolt loosening in the two-plated structure using PZT patches and presented the degree of bolt lessening by the calculated signal energy [12]. Du et al. mounted distributed PZT patches on a pipeline segment to detect the crack severity using the developed crack damage index [30].

### 2.2. Smart Aggregates

**Figure 4:**
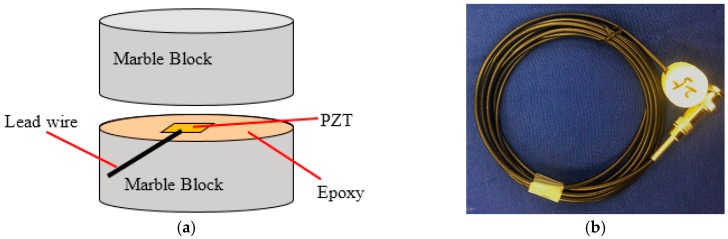
The structure of a smart aggregator: (**a**) Schematic; (**b**) A photo of SA with a BNC connector.

For the sake of protecting a fragile PZT patch which could work in concrete, a smart aggregate (SA) was designed by sandwiching a waterproofed PZT patch with two marble protections. A Bayonet Neill-Concelman (BNC) connector is made at the end of the wires to connect the SA with the sensing instrument. The structure of a SA including the schematic and the photo is shown in Figure 4. Benefiting from the extraordinary physical properties of marble material, SAs can be safely embedded in a concrete structure, and perform long-term structural health monitoring. Several applications of SAs in structural health monitoring have been reported in recent years [23,31].

## 3. Detection Principles

### 3.1. Active Sensing Approach

**Figure 5:**
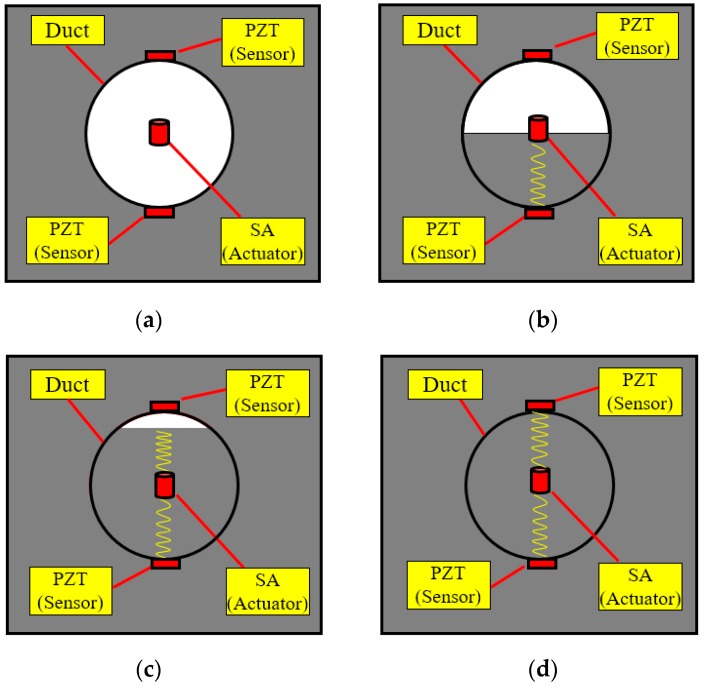
Principle of active sensing approach in monitoring of grouting compactness: (**a**) Empty duct; (**b**) Half grouting; (**c**) 90% grouting; (**d**) Full grouting.

In this research, an active sensing approach using PZT transducers is developed to monitor the grouting compactness in post-tensioning tendon duct. The schematic principle is shown in Figure 5. The SA in the post-tensioning tendon duct functions as an actuator to generate stress waves. If the duct is 100% filled with the grout, the stress wave will propagate to reach both the top and bottom surfaces of the tendon duct via the grout. However, without the presence of any grout or cement, the stress wave will fail to reach either the top or the bottom surfaces of the duct. With partial grout, the stress wave may reach or fail to reach one or both of the top or bottom surface of the duct. To enable the monitoring of the grouting compactness, two PZT sensors mounted on the outside surface of the tendon duct are used as sensors to detect the wave response. As seen in Figure 5, four different grout stages were simulated: empty, half grouting, 90% grouting, and full grouting. In order to simulate these four situations in one specimen, the grout was poured to reach each stage in an increment of five days. When the tendon duct is empty, there is no medium to allow the waves to travel from the SA to reach one of the PZT sensors. As the grout level increases towards approximately 50%, there is enough grout to allow the passage of the waves to reach from the SA to the bottom PZT sensor. At this point, however, the top PZT sensor still does not receive much signal, except for negligible amounts that may propagate through the newly poured grout to the walls of the tendon duct. The top PZT sensor will continue to receive small increases in the signal strength until the grout fully fills the tendon duct, whereupon the top PZT sensor can receive the signals generated by the SA through the poured grout. Therefore, in effect, two milestones can be inferred from the PZT sensors. In the first milestone, only the bottom PZT sensor receives the SA signal, indicating that the duct is filled by 50% grout. To reach the second milestone, both the top and bottom PZT sensors receive the signal with high strength, finally indicating that the tendon duct has been 100% filled with grout.

### 3.2. Wavelet Packet-Based Energy Analysis 

To evaluate the energy of the stress wave received by PZT sensors, the wavelet packet-based analysis is applied to provide the computed energy values of the signal received by PZT sensors. In the wavelet packet-based analysis, the sensor signal *X* can be decomposed by *n*-level wavelet packet decomposition into 2^n^ frequency bands. *X**_j_*** can be expressed in Equation (1).

*X_j_* = [*X*_*j*,1_, *X*_*j*,2_, …, *X_j,m_*] (1)

where *j* is the frequency band (*j* = 1 … 2^n^), and *m* is the amount of sampling data.

The energy E**_j_** of the decomposed signal is defined in Equation (2).

E_j_ = ||*X_j_*||^2^ = *X*_*j*,1_^2^ + *X*_*j*,2_^2^ + ··· *X_j,m_*^2^ (2)

The total energy E of the signal can be computed as the summation of all the decomposed signal energy, which is given as,

(3) $$E\;=\sum_{j=1}^{2^{n}}E_{j}$$

The energy level given by Equation (3) offers a quantitative measure of the energy of the stress wave that propagates from the SA actuator to the PZT sensors.

## 4. Experimental Setup and Procedures

### 4.1. Specimen Fabrication

**Figure 6:**
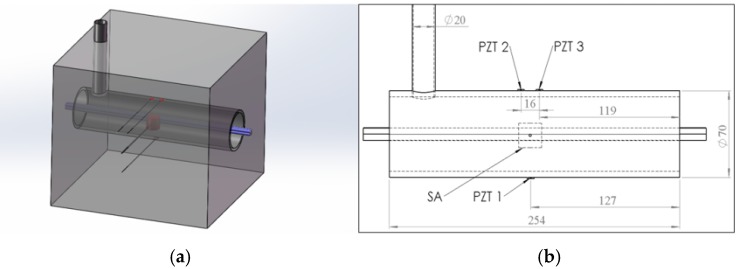
Test specimen: (**a**) the CAD model; (**b**) Schematic of the test specimen.

**Figure 7:**
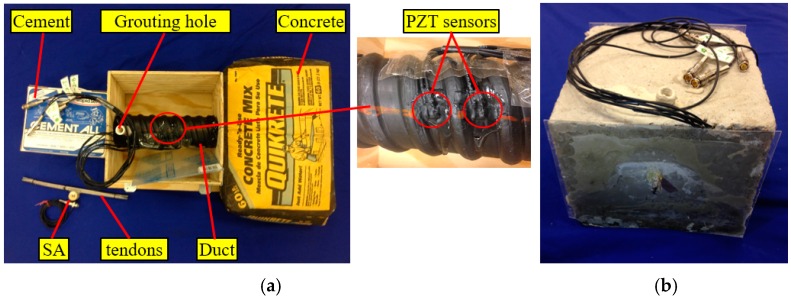
Fabricated specimen: (**a**) Ungrouted specimen; (**b**) Grouted specimen.

Figure 6 gives the CAD model, dimensions, and the sensor location for the test specimen. One SA was bonded to the tendon using epoxy and installed in the pre-determined location in the tendon duct, as shown in Figure 6a. Three waterproofed PZT sensors, including one sensor (PZT 1) mounted on the bottom outside surface and the other two sensors (PZT 2, PZT 3) mounted on the top outside surface, were used as sensors to detect stress wave, as shown in Figure 7a. The length, width, and height of the concrete specimen are all 254 mm. The diameter and thickness of the tendon duct are 70 mm and 5 mm, respectively. Detailed locations of the SA and PZT sensors are shown in Figure 6b. The test specimen used two types of binding materials i.e., concrete mix and cement, as shown in Figure 7a. The concrete mix is a 27.6 MPa blend of portland cement, sand, and gravel. The cement used for grouting in this research is a multipurpose construction material with underlayment, casting, anchoring, industrial grouting, and concrete repair. A PVC pipe was utilized as the grouting tube. Two plastic plates were attached on both sides of the tendon duct to prevent the leakage of grout from the duct during the grouting process, as shown in Figure 7.

### 4.2. Experimental Setup

**Figure 8:**
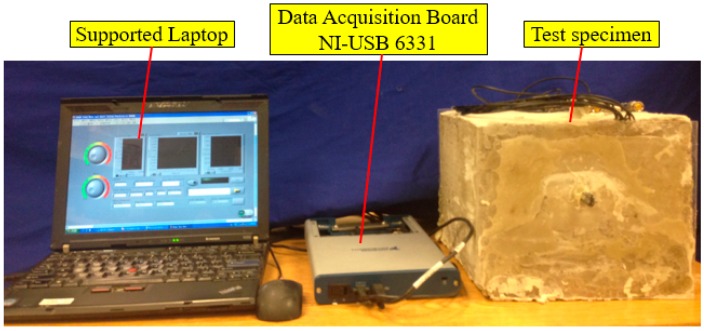
Experimental setup.

The experimental setup, including the test specimen, a data acquisition system (NI-USB 6331), and a supporting laptop, is shown in Figure 8. The data acquisition board was used to generate the signal to the SA and collected the signal response from the PZT sensors.

### 4.3. Experimental Procedures

**Figure 9:**
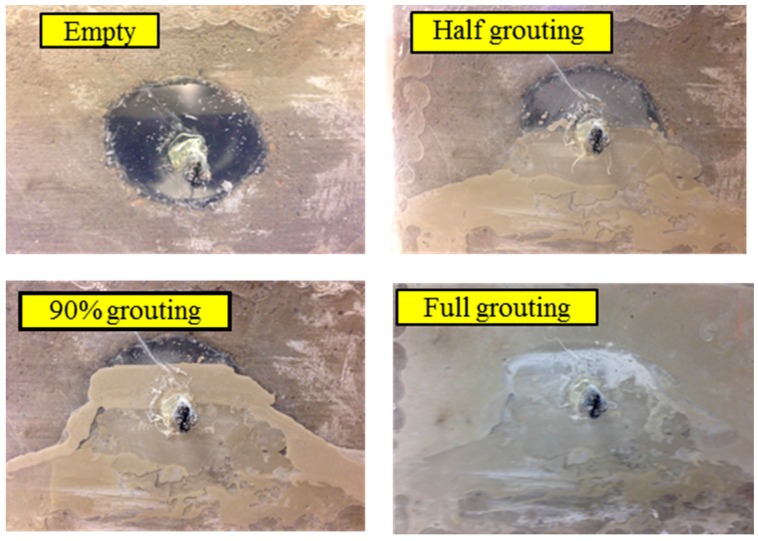
Specimen in four different grout stages.

The grouting process began 2 days after the concrete was cast. The pre-mixed cement was carefully poured through the grouting tube. The percentage of the grouting process was controlled by measuring the height of the cement level in the duct from the transparent plastic plate. The grout pouring was performed in four progressive stages: no grout (empty duct), half grouting, 90% grouting, and full grouting, as shown in Figure 9. In each stage, the grout was allowed to cure for 5 days and the proposed method was applied to monitor the status of the grout progress, before moving to the next stage. During the monitoring process, a repeated swept sine wave was applied to the PZT-based SA to generate a stress wave. Meanwhile, the signal response of each mounted PZT sensor was recorded. The frequency range of the swept sine wave is from 100 Hz to 150 kHz. The amplitude and the period of the swept sine wave is 10 V and 1 s, respectively. The sampling frequency of each channel is 2 MHz.

## 5. Test Results and Discussions

### 5.1. Time Domain Analysis

**Figure 10:**
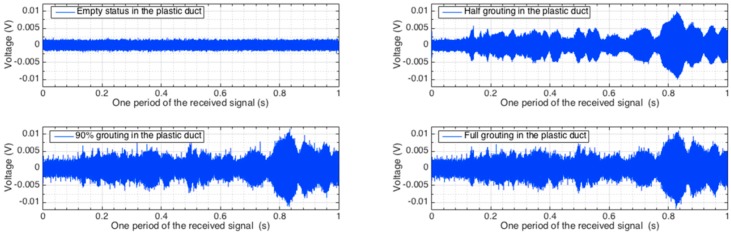
Time-domain signal of PZT 1 sensor in different grout stages.

**Figure 11:**
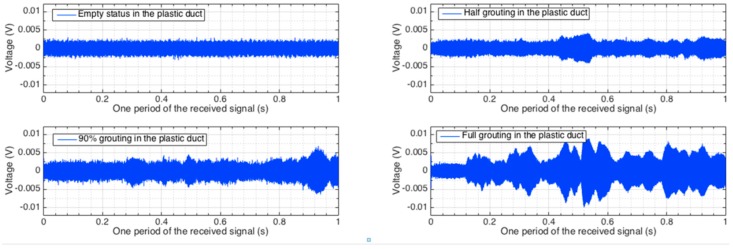
Time-domain signal of PZT 2 sensor in different grout stages.

**Figure 12:**
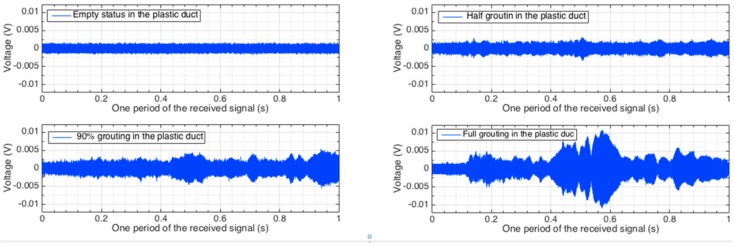
Time-domain signal of PZT 3 sensor in different grout stages.

The signals received by the distributed PZT sensors corresponding to one period of the excitation signal in all the investigated stages are shown in Figure 10, Figure 11 and Figure 12. Each figure shows the sensor signals from one of the four different grouting stages, including empty tube, half grouting, 90% grouting, and full grouting. Sensor PZT 1 mounted on the bottom surface of the tendon duct cannot detect any signal from the SA if the tendon duct is empty. The reason is that there is no medium/grout between the actuator SA and sensor PZT 1 and therefore the stress wave propagation is prevented. When the grout level increases to approximately 50%, the grout, as a conduit between the SA and PZT, allows the stress wave to propagate. Therefore, the PZT 1 sensor starts to receive a meaningful signal and the received signal remains in the two following stages. For PZT 2 and PZT 3 mounted on the top surface of the tendon duct, not much signal can be detected before the full grout stage, except for negligible amounts that may propagate through the newly poured grout to the walls of the tendon duct. When the grout completely fills in the tendon duct, the stress wave can propagate to the top side of the tendon duct. In this case, PZT 2 and PZT 3 start to receive significant signal from the SA.

### 5.2. Wavelet Packet-Based Energy Analysis

**Figure 13:**
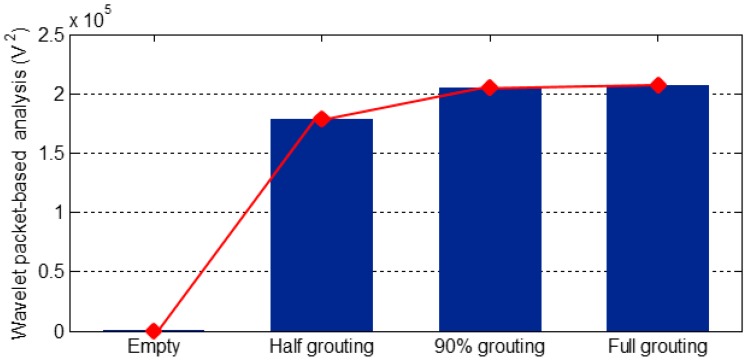
Wavelet packet-based energy analysis of PZT 1 located on the bottom surface.

**Figure 14:**
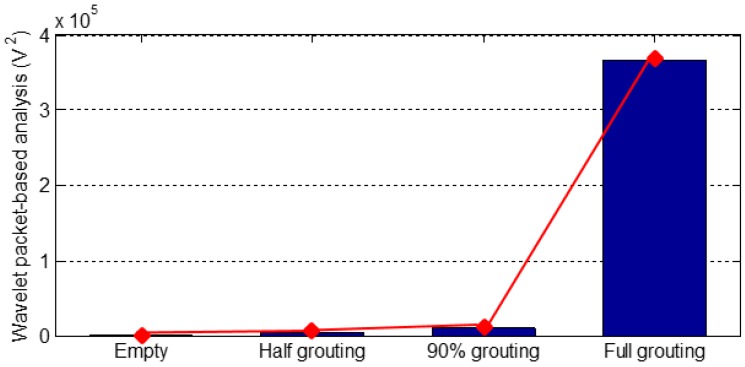
Wavelet packet-based energy analysis of PZT 2 located on the top surface.

**Figure 15:**
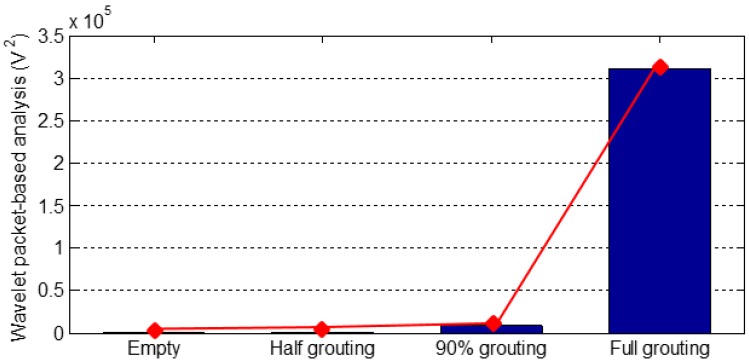
Wavelet packet-based energy analysis of PZT 3 located on the top surface.

In order to directly detect the grout situation, the energy of the received signal was computed using the wavelet packet-based energy analysis and the results are shown in Figure 13, Figure 14 and Figure 15. Figure 13 shows the energy levels of PZT 1 in all four different stages of grout pouring. Please note that the SA, as an actuator to generate the stress wave, is placed in the middle of the duct and PZT 1, as a sensor to detect any possible stress wave, is bonded on the bottom surface of the duct. When the tendon duct was empty, there was no medium/grout between the SA and PZT 1, and it is clear that the PZT 1’s energy level was zero, as shown in Figure 13. When the grout level approached approximately 50%, PZT 1 began to receive the stress wave signal and the computed energy rapidly increased from the empty stage and half grouting stage, as revealed in Figure 13. As the grouting reached 90%, Figure 13 shows that the energy level of PZT 1 further increases. We also note that the energy levels for 90% and 100% are almost the same in Figure 13, which can be easily explained by the fact that paths for the stress wave to propagate from the SA to PZT 1 are almost identical for both 90% and 100% grouting stages.

For both PZT 2 and PZT 3, which are located on the top surface of the tendon duct, there is no medium/grout between the SA and PZT 2 or PZT 3 during the empty duct, 50% grouting, and 90% grouting stages, therefore the energy levels of both PZT 2 and PZT 3 are very low for these three stages, as shown in Figure 14 and Figure 15. As the grouting level reaches 100%, a complete stress wave propagation path is formed between the SA and PZT 2 or PZT 3, and hence there is a significant increase of the energy level of signals from both PZT 2 and PZT 3, as shown in Figure 14 and Figure 15.

It is clear from Figure 13, Figure 14 and Figure 15 that the case of a strong signal from the sensor on the bottom surface and a weak signal from the top surface indicates the first milestone, i.e., 50% grouting, is achieved. The strong signals with high energy levels from the sensors bonded on both top and bottom surfaces of the tendon duct indicate that the second milestone, i.e., the 100% grouting, is achieved. In summary, by observing the sensor responses and associated energy levels, we can monitor the grouting compactness in the tendon duct in real time. In addition, the sensor responses can clearly reveal the two important milestones of 50% and 100% grouting condition.

### 5.3. Discussion

In this study, the grouting compactness in the post-tensioning tendon duct was successfully monitored in real time by using the proposed PZT based active sensing approach. Two important milestones including 50% and 100% grouting conditions can be clearly confirmed by the appearance of the strong signal received by PZT sensors. It should be noted that the amplitude of the received signal may further change with the cement hardening process since the mechanical properties of the wave propagation medium change with time. Studying the change of the sensor signal amplitude may be another interesting topic when monitoring the cement hydration progress in tendon duct, and may be considered a future work. Since the proposed method is limited to monitoring the local grouting compactness of the duct, several sets of actuators and sensors must be deployed to evaluate the general grouting compactness of large-scale structures in the practical applications. In addition, isolation and protection of the surface mounted PZT patches especially in harsh environments must be considered.

## 6. Conclusions

A practical problem associated with reinforced concrete structures with post-tensioning is the partially filled or empty tendon duct, which may reduce structural integrity and service life, and even cause accidents. This paper proposes a real-time method to monitor the grouting compactness of a post-tensioning tendon duct for reinforced concrete structures. The proposed method employs a stress wave-based active sensing approach with piezoceramic transducers. In the proposed method, one PZT based smart aggregate (SA), which was bonded on the tendon, was utilized as an actuator to generate the stress wave. Meanwhile, waterproofed PZT patches mounted on the top and bottom surfaces (outside) of the tendon duct were used as sensors to detect the stress wave. Grout in the tendon duct functions as the wave propagation medium which allows the propagation of the stress wave. If the duct is 100% filled with the grout, the stress wave generated by the SA will propagate to reach both the top and bottom surfaces of the tendon duct via the grout. However, without any grout, the stress wave will fail to reach either the top or the bottom of the duct. In addition, wavelet packet-based energy analysis was adopted in this research to compute the total signal energy received by PZT sensors. In this research, four different grout stages, including empty duct, half grouting, 90% grouting, and full grouting, were experimentally studied. Experimental results clearly demonstrate that the case of strong signals with high energy levels from the sensors bonded on both top and bottom surfaces of the tendon duct indicate the achievement of the milestone of the 100% grouting condition. In summary, by observing the sensor responses and associated energy levels, we can monitor the grouting compactness in the tendon duct in real time.
